# Supplementary material for: Antitumor Effect of Korean Red Ginseng through Blockade of PD-1/PD-L1 Interaction in a Humanized PD-L1 Knock-In MC38 Cancer Mouse Model
Source: Int J Mol Sci. 2023 Jan 18;24(3):1894. doi: 10.3390/ijms24031894 (PMC9915403; doi:10.3390/ijms24031894)
Supplement: Supplementary file 1 [file ijms-24-01894-s001.zip › ijms-2141074-supplementary.pdf]

Supplementary materials

Cell viability assay

Flow cytometry was performed to determine cell surface PD-L1 expression by 30-min incubation on ice with APC-anti-human PD-L1 (#393610, BioLegend, 1:200) and corresponding isotype control mAbs APC-anti Mouse IgG1 (#400122, BioLegend, 1:200). The cells were washed with PBS containing 2% BSA and 1mM EDTA and analysis with a Gallios flow cell counter (Beckman Coulter, Inc., Brea, CA, USA).

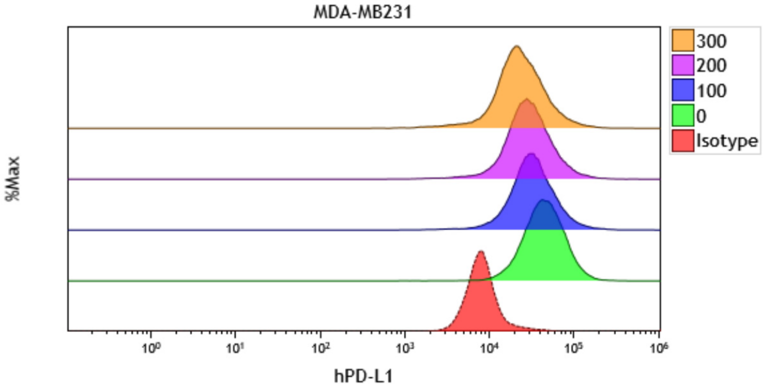

Figure S1. Effects of RGE on human PD-L1 expression by flow cytometry. MDA-MB231 cells (5×10^5 cells/ml) were treated with RGE at various concentrations (0–300 μg/mL) for 24 h and human PD-L1 expression was detected by flow cytometry.
